# KARNet: A Novel Deep-Learning Approach for Dementia Stage Detection in MRI Images

**DOI:** 10.7759/cureus.83548

**Published:** 2025-05-06

**Authors:** Wenlong Zhao, Vivens Mubonanyikuzo, Liang Zhou, Jingzhen Guo, Asad Saleem, Kaiyi Liang, Temitope E Komolafe, Tao Wu

**Affiliations:** 1 Collaborative Research Center, Shanghai University of Medicine and Health Sciences, Shanghai, CHN; 2 Rehabilitation Department, The Affiliated Zhoupu Hospital, Shanghai University of Medicine and Health Sciences, Shanghai, CHN; 3 College of Health Science and Engineering, University of Shanghai for Science and Technology, Shanghai, CHN; 4 Department of Radiology, Jiading District Central Hospital, Shanghai University of Medicine and Health Sciences, Shanghai, CHN

**Keywords:** alzheimers dementia, deep-learning, disease classification, kolmogorov-arnold network, magnetic resonance imaging, principal component analysis, resnet-18, transfer learning

## Abstract

Introduction

Accurate detection and staging of dementia are crucial for early intervention and effective patient management. Magnetic resonance imaging (MRI) serves as a valuable diagnostic tool, and deep learning models have the potential to enhance its accuracy and efficiency.

Objective

This study introduces KARNet, a novel deep-learning framework that integrates the Kolmogorov-Arnold network (KAN) architecture with a modified residual neural network (ResNet-18) and principal component analysis (PCA) to classify four stages of dementia: non-demented, very mild dementia, mild dementia, and moderate dementia.

Methods

To optimize model performance, we employ transfer learning by modifying a pre-trained ResNet-18 as a feature extractor, followed by a KAN layer as the classifier. PCA is adopted to reduce training time and computational complexity. Additionally, an ablation study and hyperparameter optimization are conducted to evaluate the robustness of the proposed model and improve performance.

Results

Experimental results demonstrate that KARNet achieves a classification accuracy of 98.5%, outperforming the existing state-of-the-art models. Evaluation on the Alzheimer's Disease Neuroimaging Initiative (ADNI) dataset confirms its effectiveness in enhancing classification accuracy and model reliability for dementia staging.

Conclusion

The findings suggest that KARNet is a promising deep-learning framework for the early diagnosis and monitoring of dementia stages using MRI, offering a potential advancement in automated dementia assessment.

## Introduction

Alzheimer's disease (AD) is a neurodegenerative disorder characterized by a progressive decline in memory, cognitive function, and behavior. As the most prevalent cause of dementia in the elderly, AD represents a significant and growing global health challenge. The prevalence of dementia is increasing at an alarming rate, with a new case being diagnosed every three seconds. It is projected that the global population of individuals living with dementia will double approximately every 20 years, reaching 78 million by 2030 and 139 million by 2050 [1]. The fastest demographic growth is occurring in regions such as China, India, and Southeast Asia, where more than 10 million new dementia cases are reported annually [2].

Accurate classification of dementia stages from MRI images is crucial for advancing clinical practice. Early-stage detection enables timely interventions that may slow cognitive decline and improve patient quality of life. Mid-stage classification assists clinicians in adjusting care strategies, managing emerging neurological symptoms, and delivering stage-specific therapies. Late-stage identification is essential for planning palliative care, supporting caregivers, and optimizing healthcare resource allocation. Therefore, precise and reliable staging across the disease progression is vital for enabling personalized treatment, informed patient counseling, and effective clinical decision-making.

Recent advancements in deep learning have driven innovation across various domains, particularly in the field of medical image analysis. These methods, which enhance algorithmic learning from data, have shown great promise in improving diagnostic accuracy and efficiency. In the case of AD diagnosis, convolutional neural networks (CNNs) and vision transformers (ViTs) have garnered considerable attention for their potential to classify and analyze complex imaging data [3-5]. CNNs are particularly effective in capturing local spatial hierarchies and features, making them well-suited for image classification tasks [6,7]. In contrast, ViTs use self-attention mechanisms to capture global dependencies, offering an advantage in handling large-scale and complex image datasets [8].

A novel architecture, the Kolmogorov-Arnold network (KAN), recently introduced in 2024 by Liu et al. [9], has shown strong performance across various deep-learning tasks. Named after mathematicians Andrey Kolmogorov and Vladimir Arnold, the KAN is designed to model complex, high-dimensional data that is challenging for traditional neural networks. The flexible architecture and adaptive activation functions of KAN enable it to better capture non-linear patterns and improve the accuracy of models working with high-dimensional datasets. Furthermore, the network’s inherent interpretability allows for deeper insights into the underlying data structures. KANs have been successfully applied to tasks such as regression, solving partial differential equations, and continual learning [10,11]. When integrated with CNNs and ViTs, KANs have demonstrated improvements in accuracy and robustness in image classification tasks, as exemplified by Cheon [12] who combined KAN with CNNs for impressive performance on datasets such as MNIST and CIFAR-10/100.

The application of convolutional operations with KAN has been found to yield positive results, particularly in datasets with high image resolution and complexity. However, network architecture design, including the reduction factor and the number of convolutions, significantly influences model performance [13]. To address the challenges of training model complexity, we propose a novel model that combines the strengths of KAN with CNNs using orthogonal transformation and principal component analysis (PCA) to reduce feature dimensionality during training. This approach aims to enhance computational efficiency and model performance while preserving critical information.

Building on the strong performance of KANs in public datasets as mentioned above, this study evaluates the effectiveness of the KAN-CNN hybrid model, specifically ResNet-18, for classifying Alzheimer's disease stages using medical imaging data, particularly MRI scans. Given KAN's interpretability and convolutional capabilities in visual tasks, we aim to investigate the performance of the model and accuracy on dementia MRI images. The proposed model, termed KARNet, combines Kolmogorov-Arnold networks with ResNet-18 and orthogonal transformation, seeking to improve robustness and accelerate model training.

In this study, we present KARNet, a novel deep-learning model that integrates KANs with ResNet-18 and orthogonal transformations. The proposed framework improves classification accuracy, efficiently handles complex high-dimensional data, and enhances model interpretability, making it suitable for tasks such as regression and continual learning. By leveraging pre-trained ResNet-18 models, KARNet significantly reduces training time and computational costs, enabling effective adaptation to specific medical datasets even with limited data availability. To the best of our knowledge, this is the first work to apply a combination of KAN and ResNet to MRI data for dementia stage classification, thereby laying the groundwork for future advancements in deep-learning-based dementia assessment and broader medical imaging applications. Furthermore, we conduct hyperparameter optimization to assess and refine the model’s performance, improving its accuracy and robustness.

Related works

This sub-section reviews some literature about recent studies that have explored various modifications of KAN in classification tasks to improve performance.

Igali and Shamoi [14] proposed a deep learning-based diagnostic method for AD by integrating KAN with fuzzy logic systems. KAN adopts learnable activation functions represented by splines, while fuzzy logic mimics human reasoning to handle uncertainty. This hybrid approach addresses challenges in interpretability, memory efficiency, and uncertainty management in image classification tasks. However, the study is limited by a small dataset and the use of a relatively simple CNN architecture (LeNet). The authors propose future work on testing with larger datasets, exploring different CNN architectures, and experimenting with alternative fuzzification techniques.

Seydi et al. [15] adopted a wavelet-based KAN for hyperspectral image classification on the MNIST dataset. Their results demonstrated the effectiveness of incorporating wavelet transforms into the KAN architecture. The study found that the best classification quality was achieved using a KAN-based transformer architecture, with the KAN mixer configured to 64 channels. The experiments were conducted with 10 epochs and a training batch size of 32, which allowed for efficient data processing and facilitated the learning of the model.

Drokin [16] investigated several modifications of convolutional KAN (CKAN) models for image classification and segmentation, focusing on regularization and hyperparameter tuning across multiple datasets, including CIFAR-10, CIFAR-100, Tiny ImageNet, ImageNet1k, and HAM10000. They redesigned U-Net-like models to incorporate Kolmogorov-Arnold convolutional layers in place of traditional convolutions. This approach achieved state-of-the-art results in segmentation tasks on biomedical datasets such as Breast Ultrasound Images dataset (BUSI), Gland Segmentation dataset (GlaS), and Centro de Visión por Computador Colon datasets (CVC). However, further refinement of these models, exploration of their applications in other domains, and investigation of additional regularization techniques are necessary to enhance performance.

Ta et al. [17] proposed FC-KAN, a variant of KAN that uses B-splines, wavelets, and radial basis functions on low-dimensional data through element-wise operations. They compared FC-KAN to multi-layer perceptions (MLPs) and other KAN variants on the MNIST and Fashion-MNIST datasets. A combination of B-splines with the difference of Gaussian functions achieved the best performance. However, further investigation is required to assess the effectiveness of FC-KAN in deeper layers, its parameter efficiency compared to MLPs, and its performance across a wider range of datasets.

## Materials and methods

Dataset description

The data for this study was sourced from Kaggle, specifically from the dataset available in Ref. [18]. This dataset comprises MRI images categorized into four groups: non-demented (50%), very mild demented (35%), mild demented (14%), and moderate demented (1%). In the preprocessing phase, various techniques were applied to optimize the model's performance on brain images, which are particularly sensitive to noise and require high-quality visibility.

The preprocessing steps included resizing, reshaping, and image rotation to standardize input dimensions. To further improve model robustness and generalization, we developed an image augmentation method incorporating techniques such as cropping, scaling, flipping, and adjustments to brightness and contrast. This augmentation process helps to simulate a wider range of real-world variations in medical images. During data processing, proper data splitting was implemented to ensure the independence of the model’s training and testing phases, with a fixed random seed of 42. All images were resized to a uniform dimension of 224×224 pixels to ensure consistency across the dataset. Normalization was also applied to adjust the pixel values to a standard scale, thereby enhancing model convergence and improving performance during training. Data normalization standardized the input feature range, preventing potential data leakage and enhancing performance. The dataset was divided into three subsets for training, validation, and testing. The training subset comprised 80% of the total dataset, which was used to fit the model. The remaining data was split equally for the validation (10%) and testing (10%) phases.

Design of the KARNet model

In this study, we employed the pretrained ResNet-18 model, a widely recognized architecture known for its efficiency and accuracy in image classification tasks. To tailor the model to our specific dataset, we fine-tuned the ResNet-18 architecture by removing its final fully connected layer, thereby allowing for modification of the model’s output layer.

**Figure 1:**
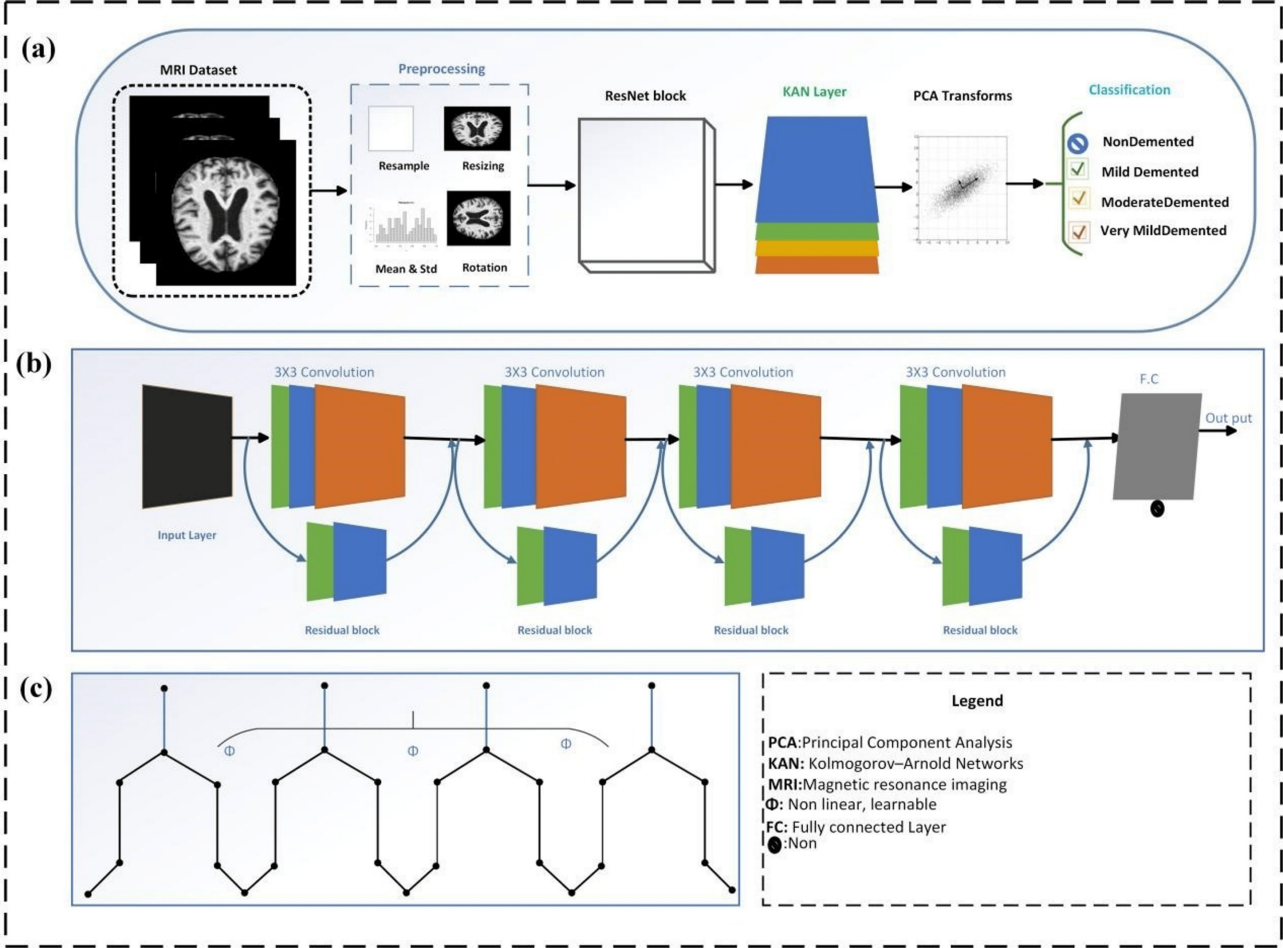
Overview of the proposed KARNet framework for MRI-based dementia classification. (a) Flow diagram showing the complete pipeline of the KARNet model, starting from MRI dataset preprocessing, feature extraction using ResNet blocks, integration of the Kolmogorov-Arnold network (KAN) layer, and dimensionality reduction with principal component analysis (PCA) transforms for final classification into non-demented, very mild, mild, and moderate dementia categories. (b) Schematic representation of the pretrained ResNet architecture used for robust feature extraction through sequential residual blocks. (c) Detailed architecture of KAN, demonstrating its structural composition for efficient dementia classification based on nonlinear, learnable functions. Image created by the authors.

Leveraging the concept of transfer learning, we incorporated a new KAN layer for classification. This adaptation enables the model to specialize in distinguishing the various stages of AD by learning the complex patterns in the MRI dataset (Figure 1). To further optimize the performance of the model and computational efficiency, we applied PCA as a dimensionality reduction technique. PCA was used to reduce the feature space complexity, thereby enhancing the overall performance and robustness of the model. This approach aims to streamline the image classification process and improve diagnostic accuracy in the multi-class classification task for AD.

The ResNet-18 architecture

In this study, we used the ResNet-18 architecture due to its computational efficiency and relatively shallow depth, making it well-suited for tasks that require a balance between performance and resource consumption. ResNet-18 is an 18-layer deep CNN that includes a combination of convolutional layers, batch normalization, and ReLU activation functions. The network was pretrained on over a million images from the ImageNet dataset [19], enabling it to learn a rich set of features useful for various image classification tasks [20].

The input to the ResNet-18 model must be an image of size 224×224 pixels, which is a standardized input size for the network. One of the key advantages of using pretrained models like ResNet-18 is the significant reduction in both training time and computational resources, as these models are capable of efficiently extracting relevant features from images across different layers.

In this study, we employ transfer learning, a powerful technique that allows us to leverage pretrained models for specific tasks, particularly when labeled data is scarce. Let us consider two domains: the source domain *D_s_* and the target domain *D_t_*. Each domain consists of a feature space *X*  and a marginal probability distribution *P*, where *X*∈*R^n^*. The source domain *D_s_* and the target domain *D_t_*  are defined as:

$D_s = X_s, P_s(X) \}, \quad D_t = \{ X_t, P_t(X)$ (1)

For each domain, there is an associated task, which typically involves a classification or regression problem. Fine-tunning involves initializing the target model with the parameters of the pretrained source model and then training it on the target task [21].

In the context of this study, we used the pretrained ResNet-18 model as the source model. The source model, trained on a large general-purpose dataset such as ImageNet, is represented by parameters. The objective is to minimize the loss function on the target task, which is achieved by fine-tuning the model on our specific dataset.

$\Theta_t = \arg\min_{\Theta} \mathcal{L}_t(\Theta)$ (2)

The optimization is performed using gradient descent [22], where the initial parameters are set to the pretrained parameters from the source model. This allows us to transfer the knowledge learned from the source domain (ImageNet) to the target domain (brain MRI images), effectively reducing the computational cost and improving the performance of the target task.

By leveraging the pretrained features of ResNet-18, the model is able to extract useful features in early layers, and the final layers are fine-tuned to specialize the classification of dementia-related brain images.

The Kolmogorov-Arnold network

KANs are an advanced class of deep learning models that offer a more flexible and powerful alternative to traditional MLPs [23]. The foundation of KANs lies in the Kolmogorov-Arnold representation theorem, which states that any multivariate continuous function can be expressed as a sum of univariate functions [24]. This theorem is crucial for KANs, as it enables the decomposition of complex multivariate functions into simpler single-variable components, enhancing the expressiveness and efficiency of the network [25].

In contrast to MLPs, which use fixed activation functions for the network’s neurons, KANs feature learnable activation functions on their edges. These activation functions are not predefined but are instead represented as univariate functions parametrized by splines. Specifically, the weight parameters in a KAN are replaced by spline functions, which allow the network to model intricate relationships between the input variables more effectively. For details on mathematical representation, see Appendix 1.

Principal component analysis

PCA, also known as the Karhunen-Loève or Hotelling transform, is a widely used linear transformation technique based on multivariate statistical methods. It is commonly applied in data analysis and pattern recognition tasks, particularly within signal and image processing [26]. PCA serves several key applications, including data compression, dimensionality reduction, and decorrelation. These functions help in managing the complexity of high-dimensional datasets by identifying and preserving the most significant variance in the data. PCA can be implemented through various algorithms, such as those based on singular value decomposition (SVD) or eigenvalue decomposition and has also been integrated with neural networks for enhanced performance [27]. In the context of image processing, PCA is particularly valuable for reducing the dimensionality of deep features extracted from higher layers of CNNs. This reduction leads to a more compact feature representation while retaining the crucial variance of the original dataset, thereby improving both computational efficiency and the effectiveness of downstream tasks such as classification or clustering [28]. For more information on mathematical representation of PCA, see Appendix 1.

## Results

Experimental parameter setup

This section presents the experimental setup and the measures used to evaluate the performance of the proposed model. The dataset is divided into three subsets: training, testing, and validation. This division ensures a robust evaluation of the model’s performance and its generalization across different portions of the data. The model is trained on a standard PC (AMD Ryzen 3 5300U with Radeon RX Vega 6 integrated graphics, 2.60 GHz). To assess the effectiveness of the training process, we selected key training parameters, including the learning rate, batch size, and number of epochs. These parameters are carefully chosen to optimize model performance and enhance its ability to generalize across the dataset. The experiment is conducted using the following settings to effectively differentiate between the various categories in the dataset. Dropout rate was set at 0.1, epochs set at 5 and increased to 10 during training, and learning rate set between 0.01 and 0. 001. The loss function used is CrossEntropyLoss, and the optimizer selected is stochastic gradient descent (SGD) with a momentum of 0.9. These configurations ensure consistent and effective training. For model validation, additional checks are performed to verify the correctness of parameter settings. For more details about parameter settings, see Table 1. 

**Table 1:** Configuration parameters for layer and grid settings

Layer configuration	in_features=initialized	in_features=initialized	-
Grid configuration	Grid_size=1	Spline_order=3	Scale_noise=0.1
Scale settings	Scale_base=1.0	Scale_spline=1.0	-
Activation function	SiLU	-	-
Grid precision	Grid_eps=0.02	-	-
Grid range	Grid range =[-1,1]	-	-

Evaluation metrics

The classification report provides key metrics for evaluating the performance of the model in classifying dementia stages in MRI images. These include accuracy, precision, recall, and F1-score:

(a) Accuracy: This represents the overall proportion of correct predictions across all dementia stages. While useful, accuracy may not fully reflect model performance when certain stages are underrepresented, such as early-stage dementia.

(b) Precision: This measures the proportion of true positives (correctly identified dementia stages) out of all instances predicted as a particular stage. A higher precision indicates fewer misclassifications between stages for example “mild” versus “moderate” dementia.

(c) Recall: It is also known as sensitivity; recall quantifies the proportion of actual positive instances correctly identified by the model. A higher recall means the model is more effective at capturing relevant cases, thus minimizing false-negatives also known as missed diagnoses.

(d) F1-Score: This precision and recall, making it particularly useful for imbalanced datasets, such as those in medical imaging. A higher F1-score indicates better overall performance in identifying and distinguishing dementia stages. These metrics offer a comprehensive evaluation of the model's performance to classify dementia stages accurately and reliably. The formulas to estimate these metrics are shown in Equations (3)-(6) below:

$\text{Accuracy} = \frac{TP + TN}{TP + TN + FP + FN}$ (3)

 $\text{Precision} = \frac{TP}{TP + FP}$ (4)

$\text{Recall} = \frac{TP}{TP + FN}$ (5)

$\text{F1-score} = \frac{2TP}{2TP + FP + FN}$ (6)

## Discussion

**Figure 2:**
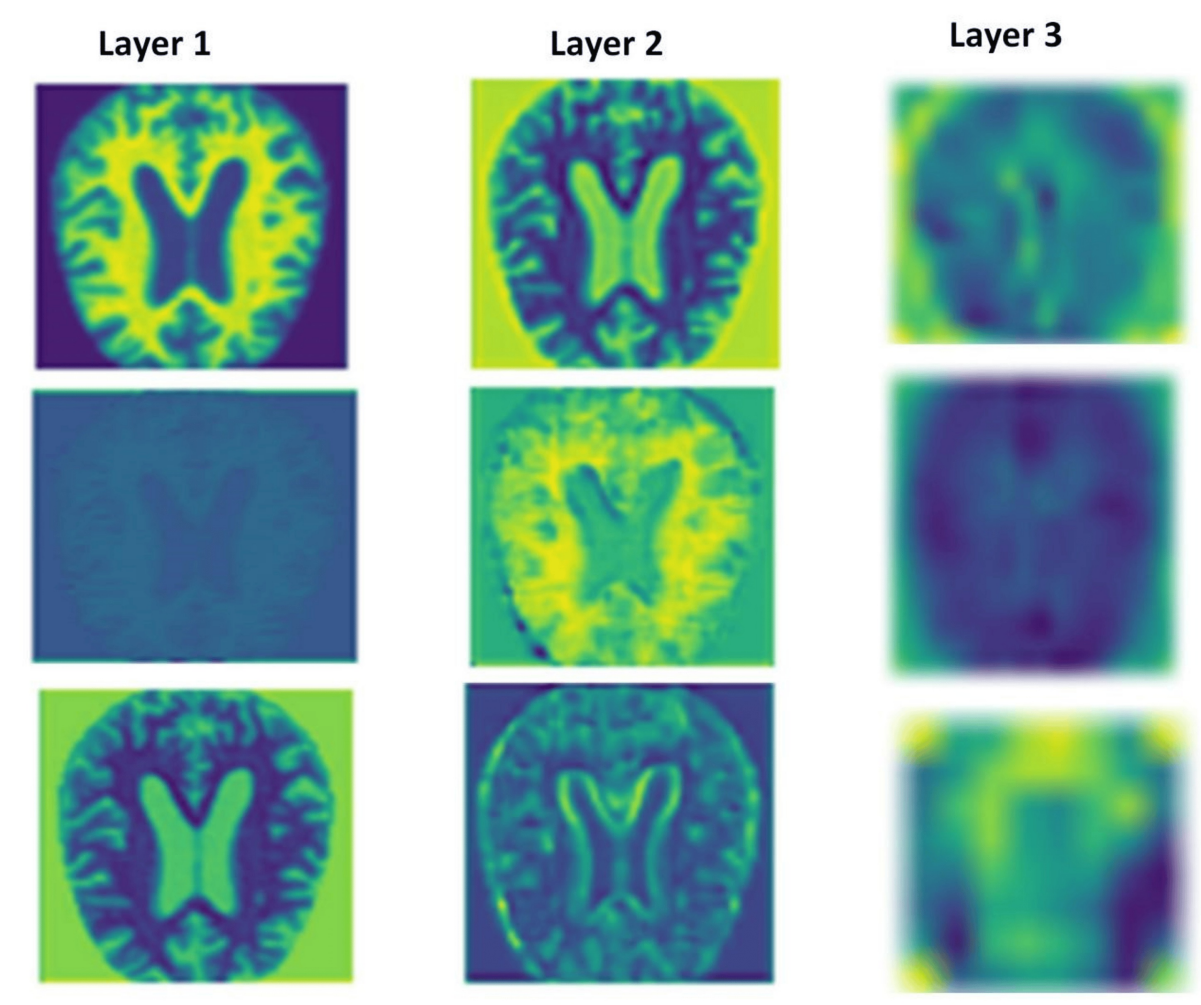
Features extracted from the first through the third layers of the ResNet model, showing the hierarchical progression of feature extraction at each layer. Image created by the authors.

**Figure 3:**
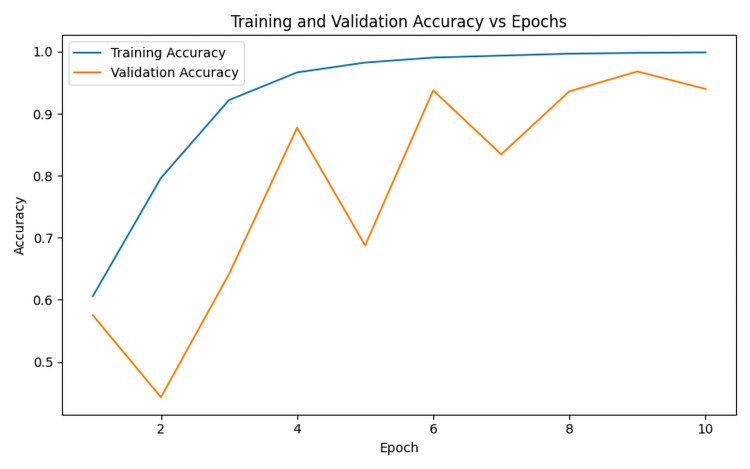
Model accuracy over 10 training epochs, illustrating the performance progression on both the training and validation datasets. Graph created by the authors.

**Figure 4:**
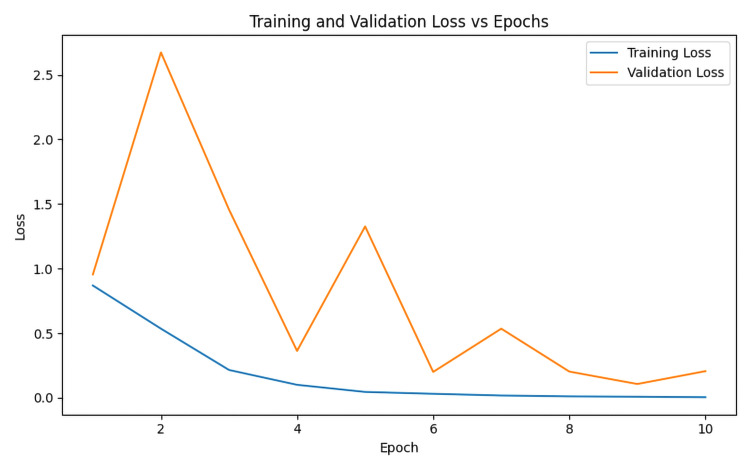
Model loss over 10 training epochs, showing the progression of both training and validation loss throughout the training process. Graph created by the authors.

**Figure 5:**
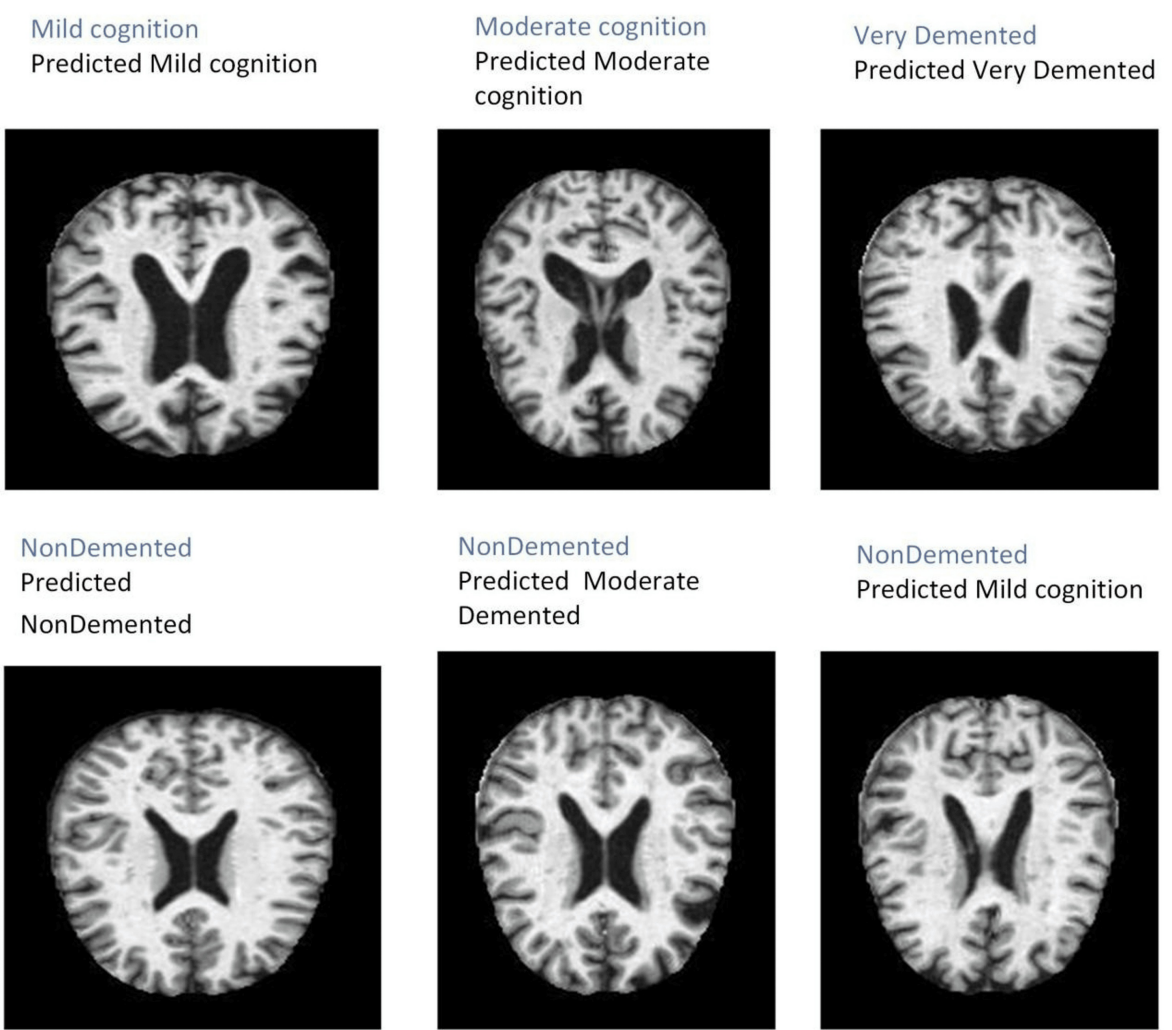
Visual display of performance of the proposed model on the test dataset for classification of dementia. This figure illustrates the generalization capability of the proposed model in predicting dementia stages on previously unseen test data. The MRI slices correspond to different cognitive states, including non-demented, mild cognitive impairment, moderate cognitive impairment, and very demented. The predicted labels indicate the classification performance of proposed model. Images generated by the authors.

**Figure 6:**
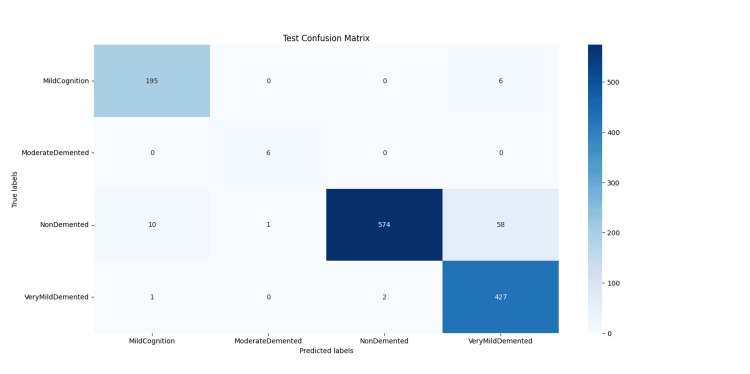
Confusion matrix for the testing dataset, showing the classification performance of the KARNet model across four dementia stages. The matrix highlights the model’s strengths in distinguishing major classes, while also indicating reduced sensitivity for the underrepresented Moderate Demented class. Matrix created by the authors.

**Figure 7:**
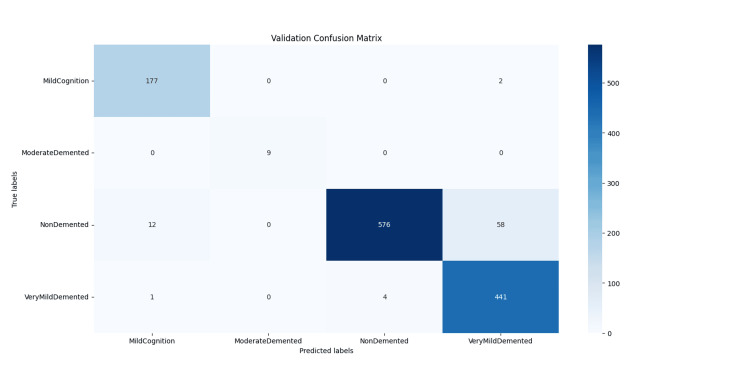
Confusion matrix for the validation dataset, showing classification performance of the KARNet model across four dementia stages. The matrix highlights true positive and misclassified cases, with notably lower detection in the underrepresented Moderate Demented class. Matrix created by the authors.

In this study, the proposed KARNet model demonstrated competitive performance when compared to state-of-the-art (SOTA) models. Our approach leveraged PCA to reduce feature complexity, thereby enhancing model efficiency. We employed a pre-trained ResNet-18 model for feature extraction, as illustrated in Figure 2, where features were extracted by the first three layers of the ResNet architecture. The model’s impressive accuracy indicates its potential as a valuable tool for early diagnosis and the development of dementia treatment strategies. As shown in Figure 3, the model achieved training and testing accuracies of 98.5% and 97.1%, respectively, underscoring its strong performance across both training and evaluation phases. Furthermore, Figure 4 highlights the model's relatively low training loss of 0.053 and validation loss of 0.450. While both losses are small, the discrepancy between training and validation loss suggests some degree of overfitting, where the model performs slightly better on the training data compared to the unseen validation data. This gap could be attributed to factors such as insufficient data or other aspects impacting generalization, which warrants further investigation. Despite this, the results indicate the model’s robust ability to generalize to unseen data, ensuring its effectiveness across diverse datasets. To further assess its real-world applicability, we evaluated the model on an unseen testing dataset. Figure 5 illustrates the model’s predictions on these unseen images. Figure 6 presents the confusion matrix demonstrating the effectiveness of the model in dementia stage classification on the test dataset, while Figure 7 illustrates the performance of the model on the validation dataset. The consistency and reliability of the results across different datasets highlight the suitability of KARNet clinical applications, particularly in the classification of AD and other stages of dementia. This reinforces the potential of our deep-learning framework as an effective tool for dementia stage detection and early diagnosis.

**Figure 8:**
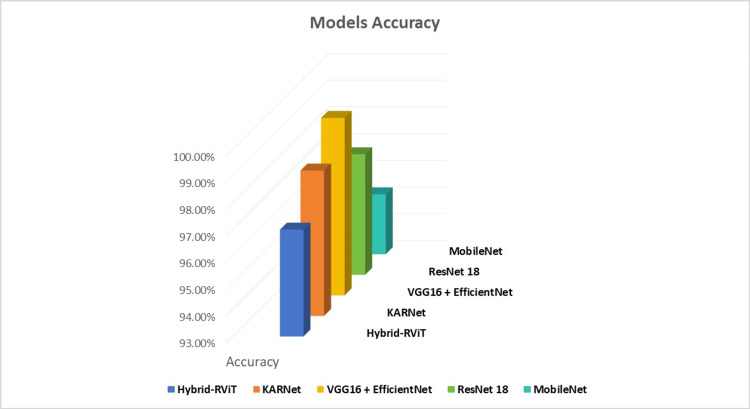
Comparison of the proposed model with state-of-the-art (SOTA) models. Chart created by the authors.

As depicted in Figure 8, our proposed model, KARNet, represents a significant advancement in the classification of dementia using MRI scans when compared to several established models, including Vision Transformers (ViT), VGG-16 (Visual Geometry Group), DenseNet-121, and Hybrid-RViT. These models have all been widely used in medical image classification tasks.

The ViT model, which applies vision transformers to structural MRI scans for AD detection, demonstrates a relatively low accuracy of 78.2%. While ViT shows reasonable precision (83.7%), its F1-score (70.4%) indicates an imbalance between precision and recall, suggesting that it may fail to detect some AD cases, potentially leading to false-negatives [28]. Moreover, the absence of recall data further limits the understanding of its effectiveness in detecting all relevant AD cases. As Alzheimer's diagnosis requires high sensitivity to ensure that no patients are missed, this limitation is a significant drawback for ViT in clinical settings.

The VGG-16 and DenseNet-121 both perform considerably better than ViT, achieving 90.15% and 87.6% accuracy, respectively. These models also demonstrate relatively balanced results, with VGG-16 showing a strong F1-score of 90.8%, indicating good harmony between precision and recall [27]. DenseNet-121 follows closely with an F1-score of 88.2%, showing solid performance in detecting AD while minimizing errors (Table 2). Despite these strong performances, both models leave room for improvement, particularly in optimizing the precision-recall trade-off. In clinical applications, reducing both false positives (incorrect diagnoses of AD) and false negatives (missed AD cases) is essential, and these models still show some weaknesses in this regard.

**Table 2:** Comparison of performance of KARNet with other methods. ViT, Vision Transformers; RViT, combination of ResNet-50 and Vision Transformer (ViT) architectures; VGG, Visual Geometry Group.

Method	Accuracy (%)	Precision (%)	Recall (%)	F1-score (%)
ViT [28]	78.2	83.7	-	70.4
VGG-16 [27]	90.15	92.1	90.2	90.8
DenseNET-121	87.6	89.3	88.4	88.2
Hybrid-RViT [5]	98.0	98.0	100	96.0
Our proposed model (KARNet)	98.5	99.0	99.0	99.0

The Hybrid-RViT model, which combines the ResNet-50 and Vision Transformer (ViT) architectures, shows notable improvements over the previous models. It achieves an impressive accuracy of 98.0% and a perfect recall of 100%, indicating that it identifies all true AD cases without missing any. This perfect recall is particularly important in clinical settings, where missing a diagnosis could have severe consequences. However, while Hybrid-RViT excels in recall, its precision (98%), and F1-score (96.0%) are slightly lower than those of VGG-16 and DenseNet-121, suggesting a higher rate of false-positives. The model’s inability to fully balance precision and recall points to a need for further refinement to ensure that both true positives and true negatives are accurately identified.

In contrast, our proposed model, KARNet, achieves exceptional performance across all key metrics for dementia stage detection. KARNet attains 98.5% accuracy, surpassing all other models in classification accuracy. More importantly, it excels in both precision (99%) and recall (99%), achieving a 99% F1-score. These results represent a significant improvement in dementia stage classification, offering minimal false-positives and false-negatives, which is critical for reducing diagnostic errors in the clinical assessment of dementia. The high recall ensures that no dementia cases are missed, while the high precision ensures that those detected are truly positive, making KARNet a more reliable tool for clinical applications. The 99% F1-score further emphasizes the model’s balanced performance, accurately identifying dementia stages while minimizing detection errors. This makes KARNet a valuable model for improving the accuracy and efficiency of dementia diagnosis and monitoring, crucial for better patient management and early intervention.

**Figure 9:**
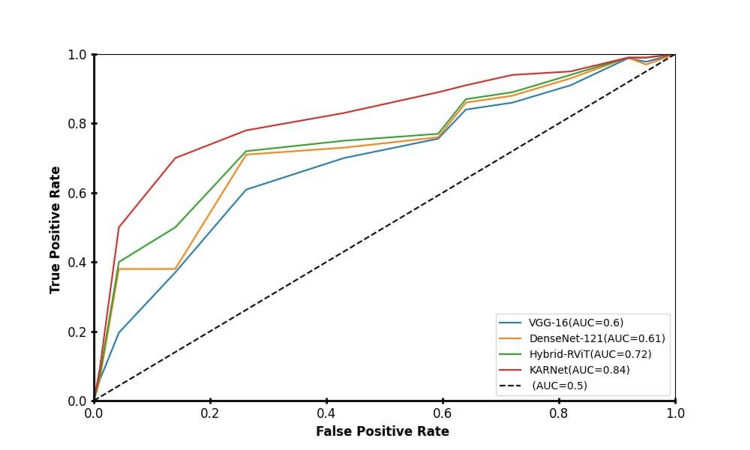
Comparison of different deep learning models using ROC curves and their respective Area Under the Curve (AUC) values. The models compared include VGG-16, DenseNet-121, Hybrid-RViT, and KARNet. A higher AUC indicates better predictive power, meaning the model is more effective at distinguishing between positive and negative classes across various classification thresholds. The proposed KARNet demonstrates the highest AUC (0.84), indicating superior performance compared to the other models. Graph created by the authors.

The graph in Figure 9 reveals that the models represented by the Hybrid-RViT and KARNet lines exhibit superior performance, as they are positioned close to the top-left corner of the ROC curve. This region corresponds to high accuracy, indicating that these models are highly effective in distinguishing between positive and negative classes. The ViT model also demonstrates commendable performance, though it is marginally less efficient than the top two models in terms of classification ability. Conversely, the VGG-16 and DenseNet-121 models are situated nearer to an area under the curve (AUC) value of 0.5, suggesting that their discriminatory power is relatively weak. These models show reduced effectiveness in distinguishing between positive and negative instances, as evidenced by their lower AUC scores, which reflect a diminished capacity to differentiate across various classification thresholds.

The superior performance of KARNet makes it a highly reliable and robust model for early dementia stage detection using MRI images. By achieving a near-perfect balance between precision, recall, and F1-score, KARNet stands out as a promising tool for enhancing dementia staging in clinical practice. The model’s ability to minimize false-positives and false-negatives ensures its effectiveness in real-world applications, where accurate, early-stage detection of dementia is crucial for timely intervention, patient care, and the management of cognitive decline. The performance of the proposed KARNet further solidifies its potential for improving diagnosis and monitoring dementia progression in healthcare settings.

Despite the promising performance of the proposed KARNet model, several limitations must be addressed in future work. First, the use of MRI data restricts the applicability of the model to patients who have access to high-quality MRI facilities, which may limit its generalizability in resource-constrained settings. Additionally, although the model performs well on the Alzheimer's Disease Neuroimaging Initiative (ADNI) dataset, its generalization to broader populations remains uncertain. Particularly, the dataset used is highly imbalanced, with only about 1% of images representing the "moderate demented" class. This imbalance may affect the ability of the model to accurately detect and generalize predictions for underrepresented stages of dementia, potentially leading to biased outcomes in real-world clinical scenarios. Addressing this issue could involve exploring advanced techniques such as data augmentation, synthetic minority oversampling (SMOTE), or cost-sensitive learning to balance class representation and enhance model fairness.

Furthermore, the performance of the model could be influenced by variations in MRI image quality and inter-rater variability in annotations, which may affect reproducibility across different clinical settings. Future research should focus on improving the model’s robustness to these variations by incorporating diverse and multi-center datasets, including other imaging modalities such as PET or CT, to increase the model’s adaptability. Moreover, potential overfitting, particularly in relation to minority classes, should be systematically mitigated through regularization techniques, ensemble learning, or uncertainty estimation methods.

Finally, while KARNet shows excellent promise, its clinical adoption will depend on seamless integration into healthcare workflows. Future studies should investigate the model’s real-time usability, scalability, and interpretability in clinical environments to ensure practical and ethical deployment.

Ablation study

An ablation study is crucial for evaluating the proposed KARNet model as it helps to determine the necessity and contribution of each component, ensuring the model’s robustness and efficacy in classifying dementia stages in MRI images. In this study, we systematically analyze key hyperparameters, including batch size, learning rate, and grid size, in relation to training epochs to understand their impact on the model’s performance. Batch size is a critical hyperparameter that significantly influences the training dynamics and convergence rate of the model. Table 3 shows the performance evaluation across different batch sizes. The results indicate that a batch size of 64 strikes the optimal balance between test accuracy (98.05%) and validation accuracy (95.78%), with minimal loss (0.1). Both smaller (32) and larger (128) batch sizes result in decreased accuracy, likely due to instability in gradient updates and suboptimal learning dynamics. This finding highlights the importance of selecting an appropriate batch size to maximize performance in detecting and classifying dementia stages.

**Table 3:** Performance evaluation of our proposed model with different batch sizes

Batch size	Test accuracy (%)	Test loss	Validation accuracy (%)	Validation loss	Important findings
32	85.47	0.6	87.50	0.5	Accuracy dropped
64	98.05	0.1	95.78	0.1	Identical performance
128	97.79	0.6	97.81	0.1	Accuracy dropped

Learning rate is another key parameter affecting the optimization process and overall model performance. We evaluated the model using three different learning rates with the SDG optimizer, as shown in Table 4. A learning rate of 0.01 yielded the highest test accuracy (98.58%) and validation accuracy (97.81%), with a minimal loss of 0.06. Lower learning rates (0.001 and 0.0001) resulted in reduced performance, with a significant drop in accuracy at 0.0001 (66.33%). This highlights that while a higher learning rate accelerates convergence, it must be appropriately tuned to avoid overshooting the optimal solution.

**Table 4:** Performance evaluation of our proposed model with different learning rates

Learning rate	Optimizer	Test accuracy (%)	Test loss	Validation Acc (%)	Validation loss	Important findings
0.01	SDG	98.58	0.06	97.81	0.1	Identical performance
0.001	SDG	90.62	0.3	89.22	0.31	Accuracy dropped
0.0001	SDG	66.33	0.7	64.77	0.71	Accuracy dropped

To examine the effect of grid size on the KAN-based model, we varied the grid size, and the results are summarized in Table 5. Increasing the grid size slightly improved the model's accuracy, with a 3% enhancement observed when transitioning from a grid size of 1 to 10. However, this improvement came at the expense of precision and F1-score, which slightly declined at the largest grid size. This suggests that while grid size adjustments can boost accuracy, the benefits may diminish beyond a certain point due to increased computational complexity or overfitting. Further investigation is needed to determine the optimal grid size for balancing accuracy and other performance metrics.

**Table 5:** Performance evaluation of our proposed model with different grid sizes

Grid size	Accuracy (%)	Precision (%)	Recall (%)	F1-score (%)	Important findings
1	90.2	91.7	91.1	91.2	Accuracy Increased
5	91.4	93.2	92.2	92.1	Accuracy Increased
10	93.3	89.6	93.8	85.2	Accuracy Increased

## Conclusions

In this study, we introduced KARNet, a novel deep learning approach that integrates ResNet-18 and Kolmogorov-Arnold networks (KAN) with PCA-based dimensionality reduction for the automatic classification of dementia stages using MRI images. The experimental results demonstrate that KARNet effectively distinguishes between multiple dementia stages, even when trained on relatively small datasets. This highlights the model’s robustness, scalability, and potential for deployment in real-world diagnostic settings, especially where access to large-scale annotated data is limited.

Future work will aim to further enhance the model’s accuracy and generalization by incorporating multimodal data sources, such as demographic and clinical variables, to provide a more holistic understanding of dementia progression. Additionally, we plan to improve the interpretability of the model by implementing explainable AI techniques, which are essential for fostering clinical trust and adoption. Addressing the current limitations, we also recommend collecting more representative data for underrepresented classes and integrating longitudinal or event-based datasets. These enhancements will help ensure that KARNet delivers clinically relevant, transparent, and reliable predictions to support informed decision-making in dementia diagnosis and care.

## Appendices

Appendix 1

Mathematical Representation of Kolmogorov-Arnold Network

Mathematically, the KAN representation theorem can be expressed as follows: Any multivariate continuous function f(x_1,x_2,…,x_n), defined over *n* variables, can be represented as a sum of continuous univariate functions: $f(x) = \sum_{q=1}^{2n+1} \left( \Phi_n \sum_{p=1}^{n} \varnothing_{qp}(x_p) \right)$                                   (1) where $\varnothing_{qp}(x_p)$ are the spline functions and $\Phi_n$ represents the transformations applied to the function. The activation function $\phi_{qp}(x_p)$ is defined as: $\phi_{qp}(x_p) = \omega(b(x)) + \text{spline}(x)$                             (2) In this expression, φ is a weight, *b*(*x*) is the basis function obtained by sigmoid linear unit (SiLU) function and spline(*x*) is the spline function. The basis function *b*(*x*) is given by: $b(x) = \text{SiLU}(x) = \frac{x}{1 + e^{-x}}$                                     (3) The spline function is parametrized as linear combinations of B-splines as shown below: $\text{spline}(x) = \sum_{n} \gamma_i B_i(x)$                                           (4) where $B_(i )(x)$ are the B-splines and γ*_i_* are their corresponding coefficients. This parametrization allows the network to model complex, non-leaner relationships by adjusting the coefficients of the B-splines during training. To assess the impact of grid size on model performance, we conducted an experiment in which we systematically increased the grid size. The grid sizes were defined in an arithmetic sequence $G_n=g_1,g_2,&hellip;,g_n$, where the *n*-th term of the sequence is given by: $g_n=A+(n-1)D$                                     (5) Here, *A* is the initial term, and *D* is the common difference between two consecutive terms. For instance, when *d*=1, we have *g*_1_=1,  *g*_5_=5,  *g*_10_=10, where *g_n_* represents the grid size at the *n*-th iteration.

Mathematical presentation of PCA

Mathematical PCA can be represented in the following steps; Calculate the input matrix X as one of the k attribute vector data where *i*=1,2,…,*n* and j=1,2,…,*m*. $X = [X_1 \;\; X_2 \;\; X_3 \;\; \dots \;\; X_n]$                                         (6) where each column $X_n$ represents an attribute vector corresponding to the *i*-th data point and

\(X_i = \begin{bmatrix} X_{i1} \\ X_{i2} \\ \vdots \\ X_{im} \end{bmatrix}\)

represents the value of the *j*-th attribute of the *i*-th data point. Then, calculate the mean $X=\bar{X}$, which statisfies the following equation: $\bar{X} = \frac{1}{2} \sum_{i=1}^{n} x_i$                                           (7) followed by estimating the covariance matrix C, which satisfies the following equation as shown: $C = \frac{1}{n - 1} (X - \bar{X})(X - \bar{X})^T$                                 (8) With covariance then compile the eigen values Ω, which satisfies the following equation shown below: $\Omega = |C - \Omega I| = 0$                                      (9) Similarly, the values of the eigen vector γ which satisfies the following equation can be estimated below: $|C - \Omega I|[\gamma] = 0$                                        (10)                         
